# A State Board of Health

**Published:** 1903-09

**Authors:** 


### EDITORIAL NOTES AND COMMENTS.

The Business office of THE JOURNAL-RECORD is No. 1007-1008 Century Building.

The Editorial office is 318-319-320 The Prudential.

Address all Business communications to Dr. M. B. Hutchins, Mgr.

Make remittances payable to THE ATLANTA JOURNAL-RECORD OF MEDICINE.

On matters pertaining to the Editorial and Original Communications address Dr. Bernard Wolff, Atlanta.

Reprints of original articles will be furnished at cost price. Requests for the same should always be made *on the manuscript.*

We will present, post-paid, on request, to each contributor of an original article, twenty (20) marked copies of THE JOURNAL-RECORD containing such article.

### A STATE BOARD OF HEALTH.

GEORGIA has at last fallen into line and the bill to establish a State Board of Health has now become a law. This is one of the many evidences of the recognition of the advance in sanitation and in preventive medicine which is due to persevering scientific work and persistent effort on the part of the medical profession to force upon the public knowledge of the importance of guarding the public health.

Such institutions exist in every State in the Union with the exception of Idaho and Arizona. It is hoped that the new institution will be fostered into a high degree of efficiency and will become one of the bulwarks of the State.

The bill is in full as follows:

A bill to be entitled an act to create a State Board of Health in and for the State of Georgia; to define its duties, jurisdictions and powers; to provide for the appointment of its members, for its organization and maintenance; to provide for its authority, and for other purposes.

SECTION 1. Be it enacted by the General Assembly of the State of Georgia, and it is hereby enacted by authority of the same, That a board to be known as the State Board of Health be, and the same is, hereby established and made one of the public institutions of the State. Said board shall consist of seven (7) members, one of whom, the secretary, shall be a member by virtue of his office, and six (6) members shall be appointed by the Governor, not less than four (4) of whom shall be physicians.

SEC. 2. Be it further enacted, That the term of office of the six (6) members first appointed shall be so arranged that the term of one of the members shall expire on the first day of January of each year for six years, and subsequent appointments shall be for the full term of six years, and any vacancy that may occur shall be filled by appointment by the Governor for the unexpired term.

SEC. 3. Be it further enacted, That the State Board of Health shall have supervision of all matters relating to preservation of the life and health of the people of the State. The board shall have supreme authority in matters of quarantine, and may declare and enforce it when deemed necessary. The board shall make and enforce reasonable orders or regulations for the prevention of the spread of contagious or infectious diseases. It shall be the duty of all local boards of health and the public and municipal officers of the State to enforce such quarantine and sanitary rules and regulations as may be adopted by the State Board of Health, and upon failure of any such officer to obey the quarantine and sanitary rules and regulations adopted by the State Board of Health, such persons shall be subject to a fine of not more than $50. The State Board of Health shall make careful inquiry as to the cause of disease, and especially when contagious, infectious, epidemic or endemic, and take prompt action to control and suppress it. It shall be the duty of the board to collect and preserve records of births and deaths and report the same, together with such other useful information, annually to the Governor. The Board of Health shall respond promptly when called upon by the State or local government and the municipal and township boards of health, to investigate and report upon the water supply, sewerage, disposal of excreta, or ventilation of any place or public buildings. The State Board of Health shall not have power to supersede municipal boards of health where the same are properly maintained, but shall act in harmony with said local boards of health. It is made the duty of the State Board of Health to enforce the provisions of chapter 3, volume 1 of the Political Code relating to the duty of the local boards of health, and the fines and forfeitures arising from the conviction of any person violating any of the laws of health and quarantine now of force in this State (or any violation of any reasonable rules and regulation for the protection of the public health of this State, promulgated by the State Board of Health) shall be paid into the treasury of the city or county where said conviction was had, and to be expended in aid of quarantine and other sanitary laws.

SEC. 4. Be it further enacted, That the said board shall have authority to make such rules and regulations as are necessary to carry into effect the scope and purpose of this act; and especially such reasonable rules and regulations for the establishment, maintenance and enforcement of quarantine regulations as the board in its discretion may deem necessary, not in conflict with the laws of the State.

SEC. 5. Be it further enacted, That it shall be the duty of the local boards of health, and of physicians in localities where there are no health authorities, to report to the State Board of Health promptly upon the discovery thereof the existence of any of the following diseases, to wit: Asiatic cholera, yellow fever, scarlet fever, smallpox, diphtheria, typhus or typhoid fever, and of such other contagious or infectious diseases as the State Board of Health from time to time may specify, and when any contagious or infectious disease shall become or threaten to become epidemic in any county, city, village or hamlet, and the local authorities shall neglect or refuse to enforce sufficient measures for its prevention, the State Board of Health may appoint a medical or sanitary officer, with such assistance as he may require, and it shall be the duty of such officer to enforce the orders or regulations of the State Board of Health.

SEC. 6. Be it further enacted, That it shall be the duty of the State Board of Health to make annual report to the Governor of the State on or before the first day of January of each year, which shall be for the preceding calendar year, and such report shall include so much of the proceedings of the board, and such information concerning vital statistics, such knowledge respecting diseases, and such instructions on the subject of hygiene as may be thought useful by the board for dissemination among the people, with such suggestions as to legislative action as it may deem necessary.

SEC. 7. Be it further enacted, That at the first meeting of the State Board of Health it shall elect two officers, a president and a secretary. The president shall be elected from the members composing the board, but no member shall be eligible to the office of secretary. The members of the board shall receive no salary, but each member shall receive $5 per day and necessary traveling and hotel expenses when on actual duty or attending the meetings of the board or pursuing special investigations for the State. Meetings of the board shall be semi-annual and at such place and time as a majority of the board may determine. The president of the board may call special meetings in case of emergency. A majority of the members shall constitute a quorum for the transaction of business.

SEC. 8. Be it further enacted, That at the first meeting of the board a secretary shall be elected by the board who shall be a competent physician, and who shall hold his office for six years, unless sooner removed by a majority vote of the entire board for failure to properly perform the duties of his office. The secretary shall receive a salary of $2,000 a year; he shall keep his office in Atlanta, and shall keep a record of the transactions of the board; he shall be the custodian of all papers, books, documents, and other property belonging to the board, and he shall perform such duties as the board may prescribe.

